# Cost-utility of an 8-month aquatic training for women with fibromyalgia: a randomized controlled trial

**DOI:** 10.1186/ar2377

**Published:** 2008-02-22

**Authors:** Narcís Gusi, Pablo Tomas-Carus

**Affiliations:** 1 Faculty of Sports Sciences, University of Extremadura, Avda. Universidad s/n, 10071 Cáceres, Spain; 2 Department of Sport and Health. University of Évora, Rua de Reguengos de Monsaraz, No. 44, 7000-727 Évora, Portugal

## Abstract

**Introduction:**

Physical therapy in warm water has been effective and highly recommended for persons with fibromyalgia, but its efficiency remains largely unknown. Should patients or health care managers invest in this therapy? The aim of the current study was to assess the cost-utility of adding an aquatic exercise programme to the usual care of women with fibromyalgia.

**Methods:**

Costs to the health care system and to society were considered in this study that included 33 participants, randomly assigned to the experimental group (*n *= 17) or a control group (*n *= 16). The intervention in the experimental group consisted of a 1-h, supervised, water-based exercise sessions, three times per week for 8 months. The main outcome measures were the health care costs and the number of quality-adjusted life-years (QALYs) using the time trade-off elicitation technique from the EuroQol EQ-5D instrument. Sensitivity analyses were performed for variations in staff salary, number of women attending sessions and time spent going to the pool. The cost effectiveness acceptability curves were created using a non-parametric bootstrap technique.

**Results:**

The mean incremental treatment costs exceeded those for usual care per patient by € 517 for health care costs and € 1,032 for societal costs. The mean incremental QALY associated with the intervention was 0.131 (95% CI: 0.011 to 0.290). Each QALY gained in association with the exercise programme cost an additional € 3,947/QALY (95% CI: 1,782 to 47,000) for a health care perspective and € 7,878/QALY (3,559 to 93,818) from a societal perspective. The curves showed a 95% probability that the addition of the water-based programme is a cost-effective strategy if the ceiling of inversion is € 14,200/QALY from a health care perspective and € 28,300/QALY from a societal perspective.

**Conclusion:**

The addition of an aquatic exercise programme to the usual care regime for fibromyalgia in women is cost effective in terms of both health care costs and societal costs. However, the characteristics of facilities (distance from the patients' homes and number of patients that can be accommodated per session) are major determinants to consider before investing in such a programme.

**Trial registration:**

Current controlled trials ISRCTN53367487.

## Introduction

Fibromyalgia (FM) is a chronic disorder of widespread pain in combination with tenderness of at least 11 of 18 specific tender points [1]. FM affects approximately 2–3% of the general population, and more than 90% of patients are female [2-4]. The average yearly cost (updated to 2005 using a 5% annual inflation) for service utilization among patients with FM is approximately € 4,500, and the societal cost is € 8,960 [5]. These costs are largely due to the frequent use of medical services such as consultations (approximately 10 per year) and medication, and the health system and societal expenses of disability from work [2,3]. Patients with FM consume health care resources to a similar extent as patients with other chronic diseases such as diabetes mellitus and hypertension [6]. Patients with FM also incur about twice the health care costs as the general population [7], and are absent from work approximately twice as much as other employees [8].

Studies reported in scientific literature have demonstrated evidence of the benefits of physical therapy on health-related quality of life and fitness [9,10]. In particular, physical exercise in warm water has been effective in short-term programmes (less than 6 months) and is highly recommended to reduce pain and minimize mechanical impact during exercise [11-15]. However, in our earlier study of patients with FM we found that most of the gains in health-related quality of life and physical fitness achieved in 12 weeks of water-based exercise were lost after a subsequent similar period of physical inactivity [11,16]. These findings suggest the need for longer programmes or maintenance programmes, but the effectiveness of such programmes remains unknown.

These programmes must be considered in light of limited health system resources. Health system managers or decision-makers frequently select the treatment strategies based on the lowest cost per quality-adjusted life-year (QALY). Cost utility is the ratio of the incremental effectiveness of one strategy compared to another (e.g. standard medical practice), and is measured in QALYs divided by the incremental cost. To our knowledge, there is no cost-utility or cost-effectiveness study of these exercise programmes for patients with FM.

Cost-effectiveness may be studied from a health service perspective by including the costs to the health care system or from a societal perspective by adding to the health care costs those borne by the patients and society. These additional societal costs include time spent, travel costs, lost work hours, etc. The approach from a health service perspective can help inform decisions about adding services to the current health care system.

The purpose of this study was to assess the cost utility of adding an 8-month, supervised, warm water exercise programme to the usual care of Public Health Service for women with FM.

## Materials and methods

### Recruitment

**Figure 1:**
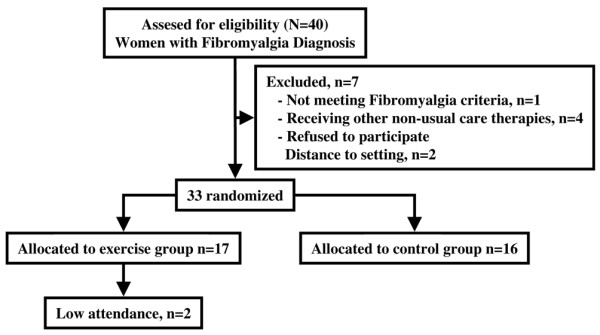
Flowchart outlining participation in the treatment.

The population of the catchment area comprised women who were in a local FM association. Eligible women were those who had FM diagnosed by a rheumatologist in accordance with the diagnostic criteria of the American College of Rheumatology (ACR) [1]. A total of 40 potentially eligible participants responded and sought further information (Figure 1). Once the study protocol was explained, 38 people gave their written informed consent. The following exclusion criteria were applied: history of severe trauma, frequent migraines, peripheral nerve entrapment, inflammatory rheumatic diseases, severe psychiatric illness, other diseases that prevent physical loading, pregnancy, participation in another psychological or physical therapy programme, or engaging in regular physical exercise more than once a week for 30 min or longer during a 2-week period in the last 5 years. The participants in our study of a 12-week aquatic programme [11] were excluded from the current trial to avoid the influence of re-training. Participants' clinical conditions were checked and a rheumatologist confirmed the diagnosis of FM. After excluding 5 candidates due to their participation in other therapies, 33 female patients, aged 37 to 71 years of age, were selected to participate. They were randomly assigned to either the exercise group (EG; *n *= 17) or a control group (CG; *n *= 16). Two patients in the EG failed to attend at least 95% of the treatment sessions due to personal reasons. Nevertheless, these patients were included in the current study to apply an intent-to-treat analysis. The trial was exclusively developed and performed at the facilities of the University of Extremadura, Spain, with the approval of the Committee on Biomedical Ethics of the University and following the updates of the Declaration of Helsinki.

### Study design

A research assistant randomized participants to either the EG or CG, according to a random number table (Table 1) and assigned a code number to each participant. Another research assistant, different from the one who supervised the treatment and analyzed data, administered the questionnaires used to gather information at baseline and after 3 and 8 months of the programme.

**Table 1:** Socio-demographic characteristics of females with fibromyalgia at baseline

	Exercise group	Control group	p Value
Age (years)^a^	50.7 (10.6)	50.9 (6.7)	0.935
Body mass index (kg/m2)^a^	28.8 (4.5)	26.6 (3.5)	0.147
Duration of symptoms (years)^a^	20.1 (8.0)	19.4 (6.9)	0.791
Number of tender points (1 to 18 points)^a^	16.9 (1.8)	17.2 (1.3)	0.563
Number of specific drugs (anti depressives, muscular relaxants, analgesics)^a^	1.3 (0.8)	1.5 (0.8)	0.379
Employment status, n (%):^b^			0.750
Blue collar	8 (53.3)	6 (40.0)	
White collar	2 (13.3)	3 (20.0)	
Unemployed	5 (33.3)	6 (40.0)	
Education level, n:^b^			0.184
Unfinished studies	1 (6.7)	1 (6.7)	
Primary school	9 (60.0)	6 (40.0)	
Secondary school	1 (6.7)	6 (40.0)	
University degree	4 (26.7)	2 (13.3)	

^a^Values expressed as mean (SD), p values from analysis of variance (ANOVA); ^b^p values from analysis of Chi-square.

### Interventions

Usual care and the addition of a water-based exercise programme were compared in the CG and EG, respectively. The usual care included standard medical attention in the public system (hospital and outpatient clinic including primary care) and the social support of the local FM association. This care could be considered the average standard of care or better for patients with FM.

The intervention added an exercise programme in a in a waist-high pool of warm water (33°C). A qualified exercise leader instructed and trained the intervention group three times a week for 1 h per session over a period of 8 months. Each session included 10 min of warming up with slow walking and easy movements of progressive intensity, 10 min of aerobic exercises at 60–65% of maximal heart rate, 20 min of overall mobility and lower limb strength exercises using water resistance, another set of 10 min of aerobics at 60–65% of maximal heart rate, and 10 min of cooling down with low intensity exercises. Heart rate was monitored using a pulse meter (Polar Accurex Plus, Kempele, Finland). During this 8-month period participants in the control group continued their daily activities, which did not include any form of physical exercise similar to that in the programme. This programme was designed without reference to any explicit behavioral model or theory, and was intended as a pragmatic intervention that could be easily organized for a large population.

### Data collection

Participants completed questionnaires, including the EuroQol EQ-5D health status instrument [17] at the beginning of the programme and after 3 and 8 months. During the same period, private and public health care was recorded, including hospital stays, drug usage, secondary and primary care appointments.

### Unit costs

The expense and time needed for travel from the patient's residence to the rehabilitation pool varied, because this facility is a scarce health resource serving a large area. To allow for a range in such additional costs, we performed two economic analyses, one from a health service perspective and another from a societal perspective. The first perspective is recommended by the National Institute for Clinical Excellence (NICE) in the UK to inform decisions on health care policy for an expensive condition. This perspective could help to decide whether to finance the addition of the programme to the health system. The second perspective is recommended to consider the combination of the burden to the patient and the health care system. The unit costs are expressed in Euros (€) based on prices in 2005.

Costs were not adjusted or discounted for changes in currency value over time, as we focused solely on effects over less than 1 year. The programme's cost was calculated based on the following: salaries at the level for a university graduate, cost of staff to run the programme, salaries at minimum wage for the patient's time (based on the 2005 official bulletin of the regional government), cost of renting a pool at a university at public prices without a grant, public bus prices, and private external management costs of the programme (insurance, monthly retrievals from patients and withdrawals to employees). Health care prices (consultations, etc.) were based on the 2005 official bulletin of the regional government. Drug prices were obtained from the Spanish version of Vademecum International [18].

### Health outcomes

The EQ-5D [17] was used to assess five dimensions of health related quality of life: (1) mobility, (2) self-care, (3) daily activities, (4) pain and discomfort, and (5) anxiety or depression. The scale for each dimension is from 1 to 3 (with 1 no problems, 2 some problems, and 3 extreme problems). Using a combination of these dimensions, a total of 243 possible health states exist. Each health state has been previously defined using the time trade-off method of utility analysis based on the response of a sample of the Spanish population [19]. This total score of utility was scaled from 1 = fully functional quality of life to 0 = death. The quality-adjusted life years (QALYs) that participants experienced over the 8-month period were estimated by calculating areas under health utility curves [20]. To avoid bias, data were adjusted by regression analysis for differences in baseline EQ-5D scores [21].

### Cost utility analysis

First, we estimated the incremental mean costs of the water-based programme and the mean QALYs added by the programme from a health care and societal perspective. Secondly, the incremental cost effectiveness ratio for the water-based programme was calculated by dividing the incremental costs by incremental QALYs.

To report the uncertainty due to sampling variation, we calculated the 95% confidence interval using the non-parametric bootstrapping technique (1,000 replicates re-sampled with replacement from treatment and control populations) and plotted a cost effectiveness acceptability curve [22,23]. This curve shows the probability that the intervention is cost effective compared with the alternative, across the range of values that decision makers are willing to pay to achieve an additional QALY. The "investment ceiling" is the level of spending that should not be exceeded, even assuming unlimited funding availability. For the health care system in Spain, the 2005 adjusted investment ceiling was set at € 34,729/QALY [24]. Decision makers should compare this upper limit of acceptable payment with estimated incremental cost effectiveness ratios to determine whether a given treatment is cost effective relative to the alternatives.

For the health system and societal perspectives, seven sensitivity analyses were performed to explore the robustness of the estimates and how dependent the results were on estimates of participants' unit costs and efficacy. From the health system perspective, the first analysis examined the influence of participation rate in the programme as this could influence the productivity by affecting the number of participants per unit of time provided by the technician. A second analysis explored the variations due to the salary changes of the technician, since this is a major source of variability in economic studies [25]. From a societal perspective, in addition to two previous analyses the third analysis estimated the cost of increasing the mean distance (in terms of time spent and the number of bus tickets purchased) from the patient's residence to the rehabilitation pool. Finally, from both perspectives, the robustness of cost effectiveness was examined by exploring scenarios combining the influence of the variations in staff salary, rate of participation, distance to the facility and effectiveness, from the lowest to the highest limit of the 95% confidence interval.

## Results

### Costs

Table 2 shows the incremental costs, to the health care system, and to society, of implementing the exercise programme. The main cost was associated with renting the pool and the difference between perspectives was mainly attributed to the cost of time spent for travel and the intervention programme. Table 3 shows the mean incremental cost per patient who participated in three sessions per week in a pool with a capacity for 20 persons. Participants in the EG and CG did not reported changes in the number of physician consultations (1 primary care visit per month; 0.3 specialist visit per month, and no hospitalizations). A total of 10 women in the EG and 5 in the CG reported changes in medication. Seven women in the EG stopped their doses of medication of amitriptyline (*n *= 7), cyclobenzaprine for sleeping (*n *= 2) or paracetamol (*n *= 1). However, two of these seven women started to take ibuprofen and another began to take cyclobenzaprine. In the CG, three women stopped the doses of medication (hydroaltesona, ibuprofen and citalopram). Over the 8 months, the weekly cost of medication increased above baseline by € 5.4 in each group as a whole; however, no remarkable incremental costs of intervention group compared to control group for medication or consultation were observed.

**Table 2:** Incremental cost of the exercise programme compared to usual care

Concept	Unit^a^	Over 8 months (€)	Total (€)
Health system costs:			
Personnel:^b^			
Sport technician	€ 9/h	1,092	
Nurse	€ 6.5/h	788	
Insurances and prevention	€ 350	350	
Facilities (renting pool and safeguards)	€ 55/h	5335	
Management	€ 24/month	192	
Medication (total health system perspective)^c^	Drug price	0	7,757
Additional societal costs:			
Time spent in therapy	€ 2.15/h	3,135	
Time spent in displacements and clothing	€ 2.15/h	3,135	
Travel costs (bus tickets)	€ 0.5/ticket	1,455	
Sub-total, societal additional costs			7,725
Total societal (additional costs and health system costs)			15,482

^a^Public cost in € in 2005; ^b^salary over 8 months = number of units × 13 h/month × 9.33 monthly salaries; ^c^no relevant incremental costs between groups were found. The weekly cost of medication increased € 5.4 from baseline in each group.

**Table 3:** Cost-utility analyses

Alternatives	Usual care	Usual care plus exercise
EQ-5D utility at baseline^a^	0.331 (0.150 to 0.511)	0.316 (0.162 to 0.470)
EQ-5D utility at 3 months^a^	0.334 (0.175 to 0.494)	0.582 (0.434 to 0.729)
EQ-5D utility at 8 months^a^	0.334 (0.175 to 0.493)	0.528 (0.380 to 0.675)
QALY over 8 months^b^	0.002 (-0.087 to 0.091)	0.133 (0.034 to 0.231)
QALY difference vs. usual care^c^		0.131 (0.011 to 0.290)
Health system perspective:		
Incremental cost/person (€)		517
Cost-utility (€/QALY)^c^		3,947 (1,782 to 47,000)
Societal perspective:		
Incremental cost/person (€)		1,032
Cost-utility (€/QALY) ‡		7,878 (3,559 to 93,818)

QALY, quality adjusted life year.

^a^Mean (95% confidence interval) estimated by analysis of covariance with adjustment for baseline EQ-5D score and then rounded to 3 significant figures; ^b^mean (95% confidence interval) using the area under the curve technique; ^c^mean (95% confidence interval estimated by bootstrapping) using the area under the curve technique.

### Health outcome

Table 3 shows that the water-based programme was associated with a greater increase in the EQ-5D utilities than the usual care during the first 3 months and this difference was preserved during the subsequent 5 months.

### Cost utility analysis

**Figure 2:**
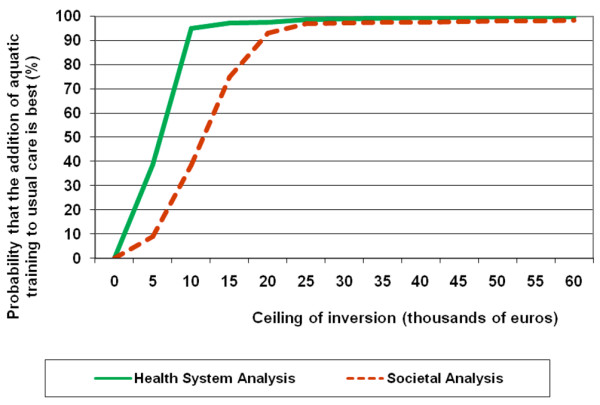
Probability curves that the addition of the aquatic training to usual care is cost-effective.

Table 3 shows the cost utility analyses from both perspectives. From the health service perspective, the Spanish Health System Efficiency Threshold was set at € 23,153/QALY for 8 months by multiplying the published threshold of 34,729 for 12 months by 8/12 [24]. From the health service perspective, each additional QALY gained by the exercise group cost in average € 3,947. However, the cost effectiveness acceptability curves (Figure 2) showed a 95% probability that the addition of the water-based programme is a cost effective strategy if the ceiling of inversion is € 14,200/QALY and a 97.5% probability if the ceiling is set at € 21,233/QALY.

From a societal perspective, the mean cost per QALY was € 7,878/QALY and there is a 95% probability that the addition of the aquatic training is cost effective if the ceiling of inversion is € 28,300/QALY. A 97.5% probability requires an inversion higher than € 42,000/QALY.

### Sensitivity analysis

The sensitivity analyses are presented in Table 4. These analyses showed the robustness of the conclusion that the water-based therapy is the best alternative compared to usual care to the variations of staff salaries and the number of participants attended per session. Nevertheless, in the worst case scenario, with a combination of minor improvements in cost utilities and a low number of clients per session, warm water exercise would be inefficient (more than € 23,000/QALY) from both perspectives. The main source of variation was observed by changing the staff salaries, effectiveness in QALYs, and the distance to the facility.

**Table 4:** Sensitivity analyses by treatment group

Manipulation of variables	Incremental cost versus usual care per person (€)	Cost utility ratio^a ^(€/QALY)
Health system analysis:		
Number of participants per pool session:		
30% lower (10 patients/group)	773	5,900
30% higher (20 patients/group)	389	2,969
Salary of personnel:		
30% lower (monitor and nurse)	479	3,656
30% higher (monitor and nurse)	554	4,229
No additional salary of nurse	465	3,550
Best case scenario of salary, participation and effectiveness^b^	348	1,200
Worst case scenario of salary, participation and effectiveness^*v*^	830	75,455
Societal analysis:		
Number of participants per pool session:		
30% lower (10 patients/group)	1,288	9,832
30% higher (20 patients/group)	904	6,901
Salary of personnel:		
30% lower (monitor and nurse)	994	7,588
30% higher (monitor and nurse)	1,069	8,160
No additional salary of nurse	980	7,481
Distance to facilities per session:		
Near (without bus ticket and 1.5 h spent)	831	6,344
Far (4 bus ticket and 2.5 h spent)	1,442	11,008
Best case scenario of salary, participation, effectiveness and near^b^	662	2,283
Worst case scenario of salary, participation, effectiveness and far^c^	1,755	159,545

QALY, quality adjusted life year.

^a^From the Spanish Health System, the cost-utility ratio threshold was set at € 23,153/QALY for 8 months in 2005; ^b^pool rental + participation 20 persons per group + QALY differential at higher limit of 95% confidence interval; ^c^salary 30% higher (monitor and nurse) + participation 10 persons per group + QALY differential at lower limit of 95% confidence interval.

## Discussion

### Principal findings

Previous studies reported the efficacy of aquatic training on patients with fibromyalgia [11-15,26,27] and the cost-utility of a 2.5 week spa treatment [28], but to our knowledge the present study is the first to report cost-utility. The major finding of this study was that the water-based programme was a cost-effective addition to usual care from both health system and societal perspectives. More precisely, an investment in this aquatic training for a similar population (sedentary women with FM) has a greater than 95% probability of being efficient according to the investment ceiling in Spain.

### Strengths and weakness

The acceptable efficiency threshold, investment ceiling or maximum willingness to pay for each gained QALY varies among countries or societies because of differences in salaries, priorities, etc. The current study applied the commonly lower threshold of € 34,729 (€ 23,153 for 8 months) used in the Spanish literature [24], but similar conclusions about the efficiency of the addition of aquatic training to usual care could be achieved using the threshold updated to year 2005 (annual inflation of 5%) often reported in American literature ($ 50,000 to $ 60,000) or Dutch literature (€ 28,940) [29].

The retention rate of patients in the our programme (88%) was similar to rates previously reported in community group-based exercise programmes in fibromyalgia (70–90%); however, aquatic training programmes usually report lower retention rates (55–75%) [9]. The social support provided by physicians, research teams, and peers with FM from the local association may have contributed to this high retention rate and the improvement in the psychosocial dimensions of health related quality of life and QALY in the exercise group. Particularly, the patient's affiliation with the local FM association brought them additional care (social support, information, etc.) in comparison to what is offered by the Public Health Care System. In this sense, the care received by the control group could be considered better than usual. By contrast, care that combines the study programme with other therapies may be even better than the programme alone. This issue could not be addressed in the current study because patients were excluded if they used other therapies (standardized behavioral or physical therapies such as massages, etc.).

The small sample size led us to use non-parametric bootstrapping techniques to treat the confidence intervals and probability curve. Health economists recommend bootstrapping techniques, rather than standard deviation-based methods, for treating the uncertainty of cost-effectiveness ratios [22,23,30-33]. The small sample, the fact that subjects were self-selected according to bioethics requirements and the catchment throughout local patient associations may limit the generalization of our findings to treatment of less motivated patients.

### Use of health care

The current study did not find any evidence for decreased use of health care services during the study period. However, the lack of change in the ratio of frequency (consultations/month) can be explained partially by the limits of supply and the management of free appointments in the general practices of the National Health System in Spain. A study in a non-limited supply setting could address the question of whether an aquatic programme could reduce the use of other health care services.

The increase in the medication cost in both groups may be partly explained because the perception of pain is slightly increased in the summer in persons with fibromyalgia [34]; with a change in the average temperature in Extremadura from 14°C at baseline to 22°C at the end of programme.

By contrast, the aquatic training in facilities with warm water was a cost-effective addition to usual care but it was not compared to other physical therapies that could reduce geographic inequalities (e.g., land-based therapies such as low-impact aerobics, walk-based exercise, tai chi, etc.) because their facilities are cheaper and easier available in more municipalities.

## Conclusion

An 8-month aquatic training programme is a cost-effective addition to the usual care provided by the Public Health System. This programme enhances the health-related quality of life in women with FM. However, the characteristics of facilities (distance from patients' homes and the number of patients that can participate per session) are major determinants that have to be considered before a health manager decides to invest in such a programme.

## List of abbreviations

CG = control group; EG = exercise group; FM = fibromyalgia; QALY = quality-life adjusted-years.

## Competing interests

The authors declare that they have no competing interests.

## Authors' contributions

NG was involved in the conception, planning and design of the study, as well as the acquisition, analysis, and interpretation of data, and writing of the manuscript. PTC was involved in the acquisition of data, analysis and assisting in the writing of manuscript. Both authors read and approved the final manuscript.
